# Circadian effects of ionizing radiation on reproductive function and clock genes expression in male mouse

**DOI:** 10.1186/s12199-021-01021-4

**Published:** 2021-10-11

**Authors:** Fenju Qin, Ningang Liu, Jing Nie, Tao Shen, Yingjie Xu, Shuxian Pan, Hailong Pei, Guangming Zhou

**Affiliations:** 1 School of Chemistry and Life science, Suzhou University of Science and Technology, Suzhou, 215009 China; 2 School of Radiation Medicine and Protection, Medical College of Soochow University, Suzhou, 215123 China

**Keywords:** Ionizing radiation, Circadian rhythm, Reproductive marker, Clock genes

## Abstract

**Background:**

Exposure to the ionizing radiation (IR) encountered outside the magnetic field of the Earth poses a persistent threat to the reproductive functions of astronauts. The potential effects of space IR on the circadian rhythms of male reproductive functions have not been well characterized so far.

**Methods:**

Here, we investigated the circadian effects of IR exposure (3 Gy X-rays) on reproductive functional markers in mouse testicular tissue and epididymis at regular intervals over a 24-h day. For each animal, epididymis was tested for sperm motility, and the testis tissue was used for daily sperm production (DSP), testosterone levels, and activities of testicular enzymes (glucose-6-phosphate dehydrogenase (G6PDH), sorbitol dehydrogenase (SDH), lactic dehydrogenase (LDH), and acid phosphatase (ACP)), and the clock genes mRNA expression such as *Clock*, *Bmal1*, *Ror-α*, *Ror-β*, or *Ror-γ*.

**Results:**

Mice exposed to IR exhibited a disruption in circadian rhythms of reproductive markers, as indicated by decreased sperm motility, increased daily sperm production (DSP), and reduced activities of testis enzymes such as G6PDH, SDH, LDH, and ACP. Moreover, IR exposure also decreased mRNA expression of five clock genes (*Clock*, *Bmal1*, *Ror-α*, *Ror-β*, or *Ror-γ*) in testis, with alteration in the rhythm parameters.

**Conclusion:**

These findings suggested potential health effects of IR exposure on reproductive functions of male astronauts, in terms of both the daily overall level as well as the circadian rhythmicity.

## Background

Outside the protection of the Earth’s magnetosphere and atmosphere is a universe full of ionizing radiation (IR). Since solar particle events (SPEs) produce a transient elevation in the rate of IR exposure in space, these events are likely to pose the greatest biological dosimetry contribution that threatens astronaut safety. Data from six SPEs (February 1956, November 1960, August 1972, August 1989, September 1989, and October 1989) have been used to model worst-case scenarios. From such models, it has been estimated that during a SPE, astronauts may be exposed to a skin dose of 14.5 Gy, testicular dose of 3 Gy, heart dose of 8 Gy, brain dose of 1.6 Gy, and lens dose of 7.5 Gy [1, 2]. These radiation doses could have clinically significant effects for the individual organs. Among various health risks induced by IR in space, fertility reduction or even permanent sterility is one of the most important barriers impeding plans for interplanetary travel [3]. As the key organ involved in maintaining male fertility, testis is a critical radiosensitive target that might be significantly impaired by IR exposure [4, 5]. The toxic effects of IR interfere with normal spermatogenesis, leading to reduced sperm counts and defective sperm production [6]. In genitourinary cancer radiotherapy, exposure to IR may result in temporary destruction or complete cessation of spermatogenesis [7, 8]. In the case of spaceflight, a recent On-orbit animal study has revealed that space radiation contributes to the degenerative changes in mouse seminiferous tubules, accompanied by a distinct downregulated sperm number and increased inflammatory level [9, 10]. It is therefore suggested that space radiation may pose health risks to the reproductive system of male astronauts.

As a result of daily changes in the environment signals (cycles in light/darkness, feeding, rest/activity, and temperature fluctuations), most organisms have developed a biological circadian clock that can synchronize physiological and behavioral processes to the 24-h light–dark (LD) cycle. At the organ level, the circadian clock also functions in the regulation of the reproductive axis consisting of the hypothalamus, pituitary, and gonads [11]. Mutations of the clock gene may impair animal fertility, and electromagnetic or chemical exposure affects the secretion of the reproductive hormone testosterone by suppressing the expression of clock genes such as *Ror*-α, *Bmal1*, and *Clock* [12, 13]. Additionally, a dose-dependent circadian phase-advancing effect has been observed in radiation-exposed organs and cells, indicating an effect of radiation on circadian rhythmic output [14–16]. However, whether IR exposure affects the diurnal rhythms of male reproductive function and its underline mechanism has not yet been investigated. In this study, we report the effects of IR on reproductive functions and their daily rhythms in terms of clock genes expressions in testis, to try to reveal chronotoxic characteristics of space IR on male astronauts.

## Materials and methods

### Animals and irradiation procedure

C57BL/6 wild-type male mice (6 weeks old) used in this study were provided by the laboratory animal center of Soochow University. The animals were maintained in strict accordance with the Institutional Animal Care and Use Committee guidelines of the University (the number approved by the committee, A201804134). They were housed in the specific pathogen-free barrier system facility maintained at 25±2 °C temperature, 50±5% relative humidity and 12 h L-D cycles (6:00 a.m. to 18:00 p.m.). Incandescent lamps (150–200 lux) were arranged at cage level to provide light during the light cycle. All animal handling operations during the dark cycle were carried out with the use of dim red light (0.1 lux). The animals were fed on commercial diet and water was provided ad libitum. Animals’ circadian rhythmicity was screened by locomotor activity after adaptive feeding.

**Fig. 1:**
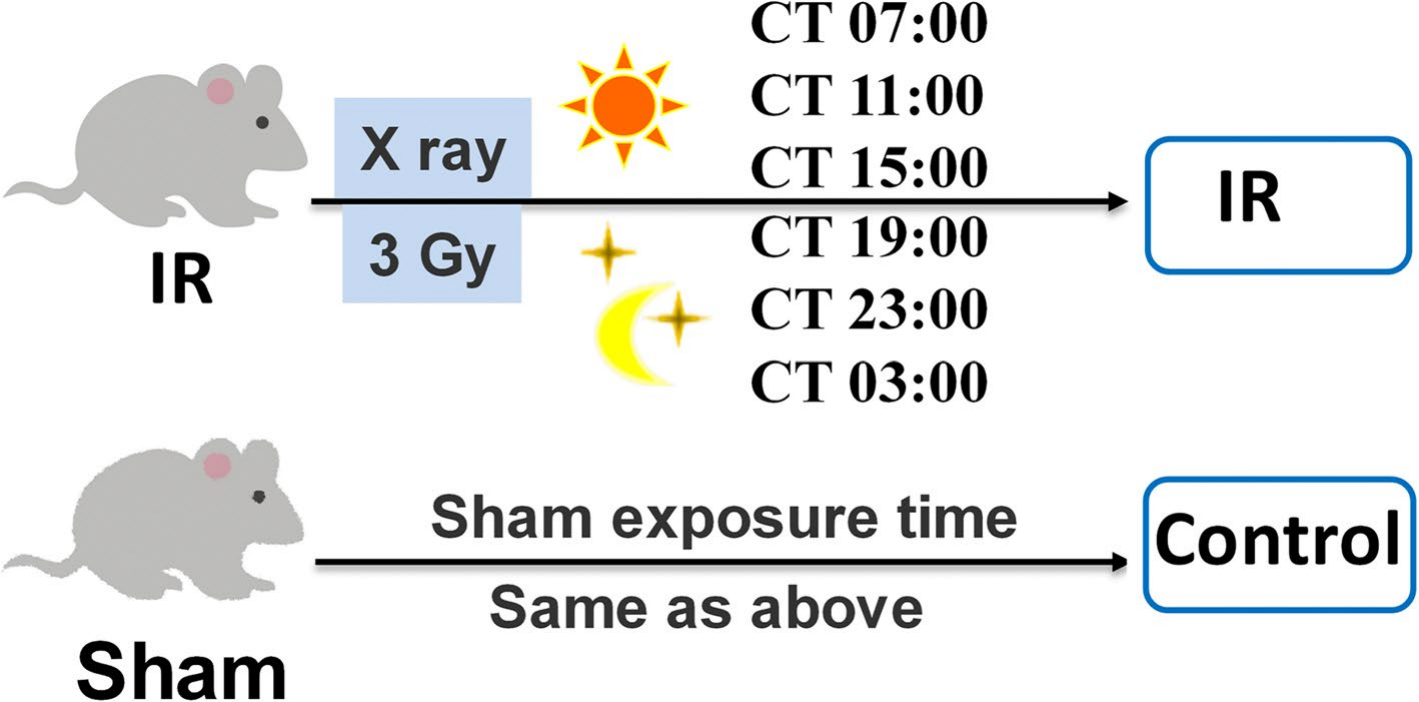
Procedure of ionizing radiation for mice

Male mice with circadian rhythmicity were randomly divided into 6 ionizing radiation (IR) groups (X-ray exposure, 36 animals) and 6 control groups (sham-exposed mice, 36 animals). Each group of mice was treated at a specific time-point of a day, and the procedure of irradiation was carried out following Fig. 1. The experimental groups were positioned in a chamber fixed to subjected to whole-body irradiation of 3 Gy X-rays at different Control Time (CT) hours: CT 3:00, CT 7:00, CT 11:00, CT 15:00, CT 19:00, and CT 23:00, respectively, by using a cabinet X-ray generator (Faxitron, Wheeling, IL, USA) operated at 100 kVp and 5 mA with a dose rate of 1.2 Gy/min. The thickness of the beryllium filter used in the X-ray generator was 0.25 mm. Mice in the six control subgroups were handled in the same manner as those in radiation-exposed groups but without X-ray. The mice were sacrificed under anesthesia by an intraperitoneal (i.p.) injection of chloral hydrate after the treatment [17]. Testes and epididymis were collected from each animal to record the weight. The right testis was examined for the testosterone levels and activity of testicular enzymes glucose-6-phosphate dehydrogenase (G6PDH), lactic dehydrogenase (LDH), sorbitol dehydrogenase (SDH) and acid phosphatase (ACP), while the left testis was used for daily sperm production (DSP) and clock gene expression detection.

### Measurement of testosterone, G6PDH, SDH, LDH, and ACP

From each mouse, the right testis was homogenized and centrifuged in saline (0.9% NaCl containing 0.05% Triton X-100) (Sigma-Aldrich, Shanghai, China), and the supernatant was used to determine the concentration of testosterone by a radioimmunoassay ELISA kit (Shanghai Yili Biotechnology Co. Ltd., Shanghai, China). A portion of the supernatant was used to measure the activities of G6PDH, SDH, LDH, and ACP by a commercially available kit (Nanjing Jiancheng Bioengineering Institute, Nanjing, China).

### Measurement of sperm production and motility

The left epididymis of each mouse was excised and minced in a petri-dish in 2 ml of warm Hank’s solution (calcium and magnesium free) to obtain a free sperm suspension. The motility of sperm was recorded in forms of (a) rapid, progressive, and linear; (b) sluggish, progressive, and linear; (c) non-progressive; and (d) no mobility (WHO, 1992).

Daily sperm production (DSP) was determined as described by Joyce KL et al. (1993) [18]. Briefly, decapsulated testes were homogenized in ice-cold 0.9% NaCl solution containing 0.01% Triton X-100. The homogenate was allowed to settle for 1 min and was gently mixed and stored on ice. The number of sperm heads was counted using a hemocytometer, and the number of spermatozoa produced per gram of testicular tissue per day was calculated using the following formula: Average count of sperm heads from four chambers × ×square factor × ×hemocytometer factor × ×dilution factor divided by testis weight (g) and the time (4.84 days) during spermatogenesis [19].

### mRNA expression of clock genes

The other testis tissue was used to extract total RNA (high Pure RNA kit, Hoffmann-La Roche Ltd, Basel, Switzerland) and the concentration was determined by the absorbance value at 260 nm. The reverse transcription of 1 mg RNA was carried out using the M-MLV reverse transcriptase Kit (Invitrogen Corporation, Carlsbad, CA). After incubation at 37 °C for 50 min, M-MLV reverse transcriptase was inactivated by heating at 70 °C for 15 min and the genomic DNA were degraded by DNase I (Beyotime Inc., Nantong, China). Real-time PCR was performed utilizing the 7500 Real-Time PCR System (Applied Biosystems Inc., Foster City, CA) and FastStart SYBR Green Master (Hoffmann-La Roche Ltd, Basel, Switzerland). In 10 ml 2 × ×Fast Start SYBR Green Mix we added 10pM of each primer set for *Clock*, *Bmal1*, *Ror-α*, *Ror-β*, or *Ror-γ* clock genes. The sequences of the primers used are shown in Table 1. The Real-time PCR was carried out in a AB step one plus (AB) using the following thermal profile: initial PCR activation step at 95 °C for 2 min, 45 cycles of denaturation at 95 °C for 20 s, and annealing and extension at 60 °C for 40 s. The fold-change for mRNA in mouse was calculated using the 2^-ΔΔCt^ method. β-actin was used as an internal control. All samples were tested in triplicates.

**Table 1:** The sequences of primers used in real-time PCR

Gene		Primer sequence 5′ to 3′
*Clock*	Forward:	CGGCGAGAACTTGGCATT
	Reverse:	AGGAGTTGGGCTGTGATCA
*Bmal1*	Forward:	GCAGTGCCACTGACTACCAAGA
	Reverse:	TCCTGGACATTGCATTGCAT
*ROR-α*	Forward:	CCACCTACTCCTGTCCTCGTCAG
	Reverse:	CTTCTGCACCTCGGCGTACAAG
*ROR-β*	Forward:	TGAGAATGTGTCGTGCCTTCAACC
	Reverse:	CAGAACAGCAGAGGAGAACAGAGC
*ROR-γ*	Forward:	GTCCAGACAGCCACTGCATTCC
	Reverse:	TGCCGTAGAAGGTCCTCCAGTC
*β-actin*	Forward:	GCTCTTTTCCAGCCTTCCTTCTTG
	Reverse:	GTGTTGCATAGAGGTCTTTACGG

### Statistical analysis

Data presented in figures are mean ± standard error. One-way analysis of variance (ANOVA) and the F-test were used to determine significant difference between groups. *p*<0.05 was considered statistically significant. Cosinor analysis was applied to fit data using the cosine software (Departments of Biology, School of Vhemistry and Life Science, Suzhou University of Science and Technology, Suzhou, Jiangsu, China). The *F*-test was used in the zero-amplitude test to analyze the significance of the circadian rhythm [20].

The data obtained were then fitted by the least squares method for cosinor analysis as expressed in the following Eq. (1).

1 $$F(t)=M+A\cos \left(\omega t-\varphi \right)$$

where *F* stands for a fitted cosine function, *M* is the median of the rhythm, *A* represents the amplitude of the rhythm, *ω* is the radial frequency (15 °/h), and *φ* is the peak phase of the rhythm.

## Results

### Circadian rhythms of reproductive markers and clock gene expressions

The data of reproductive markers such as testicular enzymes activities and clock gene expressions were shown in Tables 2, 3, and 4, respectively. The parameters of cosinor analysis (*M*, *A*, *ω*, and *φ*) were also shown in these tables.

**Table 2:** Effects of IR exposure on index levels and rhythm parameter of male reproductive function

Reproductive index	Groups	CT 3:00	CT 7:00	CT 11:00	CT 15:00	CT 19:00	CT 23:00	***M***	***A***	***ω***	***ϕ***
**Daily sperm production** **(10**^**6**^/(g*d))	**Control**	**14.16±1.53**	**18.18±2.24**	**9.80±1.01**	**8.45±0.97**	**4.88±0.37**	**11.68±1.06**	**11.19**	**5.52**	**15**	**81.63**
	**IR**	8.75±0.98*	9.33±0.99*	17.09±2.13*	25.50±2.45*	26.73±3.19*	20.72±2.32*	18.02*	9.95*	**15**	**262.60**
**Sperm motility** **(%)**	**Control**	**73.02±7.45**	**75.64±5.72**	**64.51±5.58**	**58.51±4.97**	**51.01±6.20**	**63.79±5.51**	**64.41**	**11.46**	**15**	**84.66**
	**IR**	31.53±3.79*	46.60±5.08*	28.39±4.31*	20.47±2.11*	18.70±2.34*	20.18±2.76*	27.64*	**12.54**	**15**	101.25*
**Testosterone** **(ng/ml)**	**Control**	**8.04±0.68**	**9.14±0.91**	**7.68±0.82**	**7.11±0.81**	**5.90±0.72**	**7.60±0.81**	**7.58**	**1.27**	**15**	**93.75**
	**IR**	**8.02±0.91**	**8.20±0.88**	**7.67±0.78**	**6.99±0.83**	**6.81±0.34**	**7.67±0.91**	**7.56**	0.70*	**15**	**79.99**

Note: Date are means ± standard error for DSP, sperm motility, and testosterone levels. Asterisks indicate a significant difference from control at *p* < .05 at different Control Time (CT) hours. The data obtained were then fitted by the least squares method for cosinor analysis, *M* is the median of the rhythm, *A* represents the amplitude of the rhythm, *ω* is the radial frequency (15 °/h) and *φ* is the peak phase of the rhythm. The experimental mice were subjected to whole-body irradiation of 3 Gy X-rays at different Control Time (CT) hours: CT 3:00, CT 7:00, CT 11:00, CT 15:00, CT 19:00, and CT 23:00, respectively. Control mice were handled in the same manner as those in radiation-exposed groups but without X-ray. *IR*, ionizing radiation

**Table 3:** Effects of IR exposure on the activities and rhythm parameter of testicular enzymes

Testicular enzymes	Groups	CT 3:00	CT 7:00	CT 11:00	CT 15:00	CT 19:00	CT 23:00	***M***	***A***	***ω***	***ϕ***
**G6PDH** **(nmol/min/mg prot)**	**Control**	**16.65±1.91**	**36.48±3.73**	**45.75±3.11**	**38.81±2.82**	**29.15±3.43**	**19.43±2.03**	**31.04**	**14.34**	**15**	**182.37**
	**IR**	**15.68±1.87**	21.00±2.39*	**50.72±6.31**	**41.22±2.92**	**25.18±2.26**	**22.71±2.16**	**29.42**	**15.49**	**15**	**198.64**
**SDH** **(nmol/min/mg prot)**	**Control**	**23.34±2.32**	**22.62±2.28**	**10.69±1.11**	**10.70±0.71**	**28.68±2.36**	**40.17±3.40**	**22.70**	**13.08**	**15**	**353.35**
	**IR**	13.37±1.09*	8.61±0.94*	**11.60±1.07**	**13.00±1.46**	**24.97±2.43**	24.03±2.31*	15.93*	8.33*	**15**	**311.34**
**ACP** **(μmol/min/mg prot)**	**Control**	**7.96±0.82**	**7.29±0.61**	**10.97±1.20**	**11.57±1.17**	**13.18±1.46**	**12.62±0.75**	**10.60**	**2.90**	**15**	**273.68**
	**IR**	**9.22±0.66**	2.19±0.21*	3.93±0.42*	5.14±0.35*	**13.63±1.28**	22.22±1.02*	**9.39**	8.94*	**15**	331.25*
**LDH** **(nmol/min/ml port)**	**Control**	**3.31±0.36**	**2.20±0.17**	**5.05±0.43**	**5.30±0.55**	**12.34±1.28**	**8.98±0.60**	**6.20**	**4.40**	**15**	**292.33**
	**IR**	**3.44±0.35**	**2.67±0.24**	**4.81±0.25**	**4.94±0.37**	6.61±0.41*	4.98±0.71*	**4.58**	1.64*	**15**	**271.45**

Note: Date are means ± standard error for the activities levels of testicular enzymes including G6PDH, SDH, ACP and LDH. Asterisks indicate significant difference from control at *p* < .05 at different Control Time (CT) hours. The data obtained were then fitted by the least squares method for cosinor analysis, *M* is the median of the rhythm, *A* represents the amplitude of the rhythm, *ω* is the radial frequency (15 °/h) and *φ* is the peak phase of the rhythm. The experimental mice were subjected to whole-body irradiation of 3 Gy X-rays at different Control Time (CT) hours: CT 3:00, CT 7:00, CT 11:00, CT 15:00, CT 19:00, and CT 23:00, respectively. Control mice were handled in the same manner as those in radiation-exposed groups but without X-ray. *IR*, ionizing radiation

**Table 4:** Effects of IR exposure on mRNA expression and rhythm parameter of clock genes

Clock genes index	Groups	CT 3:00	CT 7:00	CT 11:00	CT 15:00	CT 19:00	CT 23:00	***M***	***A***	***ω***	***ϕ***
***Clock*** **(Relative mRNA expression)**	**Control**	**1.00±0.01**	**1.56±0.17**	**0.90±0.01**	**0.25±0.00**	**0.44±0.01**	**0.82±0.06**	**0.83**	**0.55**	**15**	**84.34**
	**IR**	1.38±0.12*	**1.73±0.12**	0.62±0.04*	**0.24±0.03**	0.23±0.02*	**0.60±0.06**	**0.80**	**0.77**	**15**	**79.77**
***Bmal1*** **(Relative mRNA expression)**	**Control**	**1.00±0.11**	**1.45±0.12**	**0.67±0.05**	**0.21±0.02**	**0.03±0.00**	**0.45±0.03**	**0.63**	**0.66**	**15**	**90.51**
	**IR**	0.29±0.02*	0.46±0.05*	0.23±0.02*	0.11±0.01*	0.09±0.01*	0.19±0.02*	0.23*	0.16*	**15**	**90.76**
***ROR- a*** **(Relative mRNA expression)**	**Control**	**1.00±0.10**	**0.19±0.02**	**0.62±0.06**	**1.99±0.16**	**4.15±0.49**	**1.49±0.13**	**1.58**	**1.63**	**15**	**283.80**
	**IR**	**1.23±0.12**	0.28±0.02*	0.19±0.02*	0.95±0.10*	3.02±0.23*	**1.67±0.18**	**1.22**	**1.22**	**15**	**309.53**
***ROR-β*** **(Relative mRNA expression)**	**Control**	**1.00±0.09**	**0.32±0.02**	**0.83±0.08**	**2.38±0.25**	**6.37±0.43**	**3.03±0.32**	**2.32**	**2.62**	**15**	**290.16**
	**IR**	2.07±0.21*	1.70±0.13*	0.12±0.01*	0.31±0.02*	1.01±0.07*	**3.55±0.31**	1.46*	1.50*	**15**	13.11*
***ROR-γ*** **(Relative mRNA expression)**	**Control**	**1.00±0.88**	**0.38±0.04**	**0.20±0.02**	**1.96±0.13**	**3.49±0.32**	**2.10±0.22**	**1.52**	**1.54**	**15**	**295.12**
	**IR**	0.41±0.04*	0.01±0.00*	0.44±0.03*	1.11±0.09*	2.31±0.27*	0.79±0.05*	0.84*	0.95*	**15**	**278.88**

Note. Date are means ± standard error for the mRNA expression of clock genes including *Clock*, *Bmal1*, *Ror-a*, *Ror-β*, and *Ror-γ*. Asterisks indicate a significant difference from control at *p* < .05 at different Control Time (CT) hours. The data obtained were then fitted by the least squares method for cosinor analysis, *M* is the median of the rhythm, *A* represents the amplitude of the rhythm, *ω* is the radial frequency (15 °/h) and *φ* is the peak phase of the rhythm. The experimental mice were subjected to whole-body irradiation of 3 Gy X-rays at different Control Time (CT) hours: CT 3:00, CT 7:00, CT 11:00, CT 15:00, CT 19:00, and CT 23:00, respectively. Control mice were handled in the same manner as those in radiation-exposed groups but without X-ray. *IR*, ionizing radiation

**Fig. 2:**
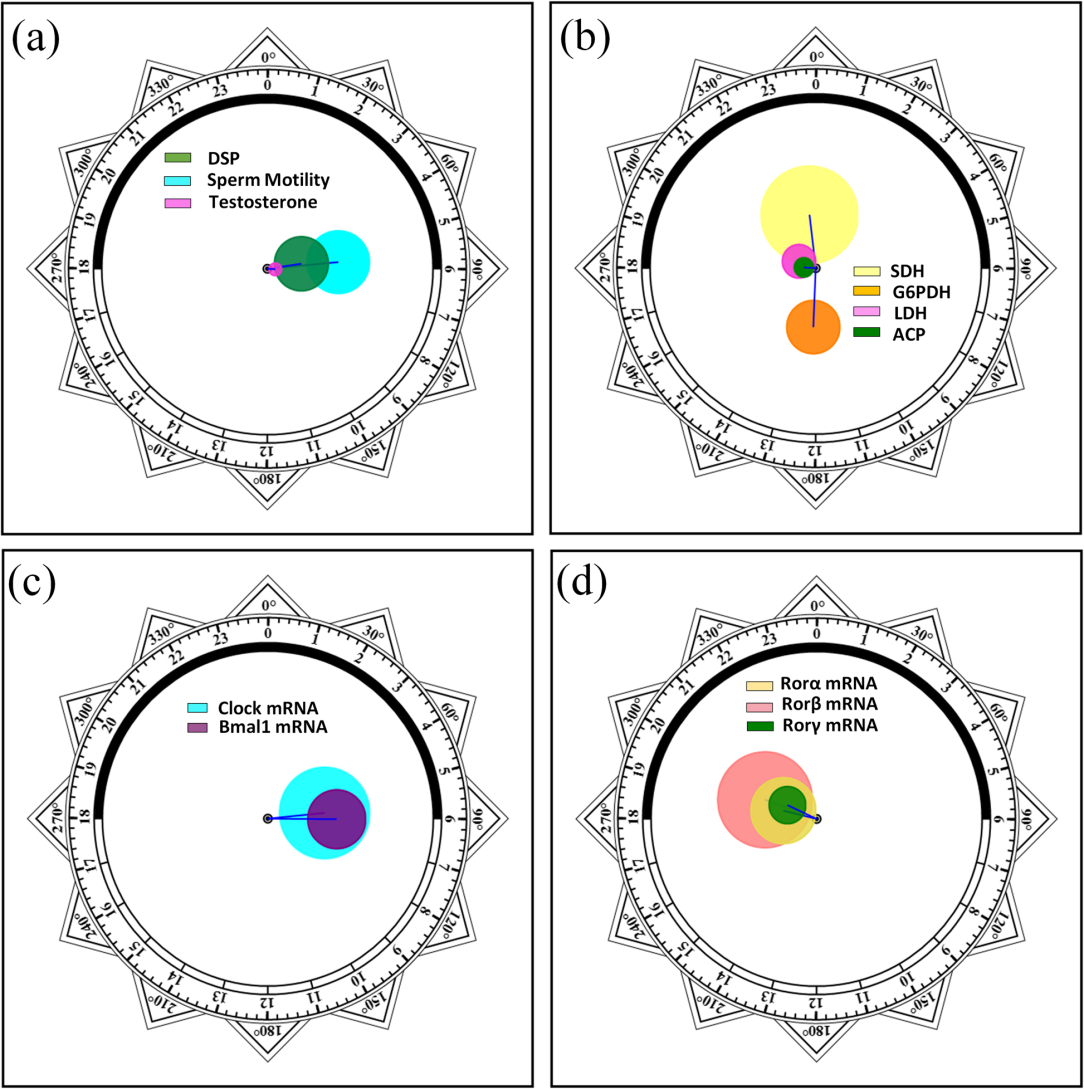
Zero-amplitude test results for circadian rhythms in unirradiated control mice. **a** DSP, sperm motility, and testosterone; **b** G6PDH, SDH, ACP, and LDH; **c** Clock and Bmal1; and **d** Ror-α, Ror-β, and Ror-γ. The circles represent the 95% joint confidence intervals of amplitude and acrophase. The pole (zero-point) of the polar coordinate was not overlapped by the circles, namely, the zero amplitude hypothesis was rejected because of *p* < 0.05, which indicates a distinct circadian rhythmicity of these reproductive markers and five clock genes expression

**Fig. 3:**
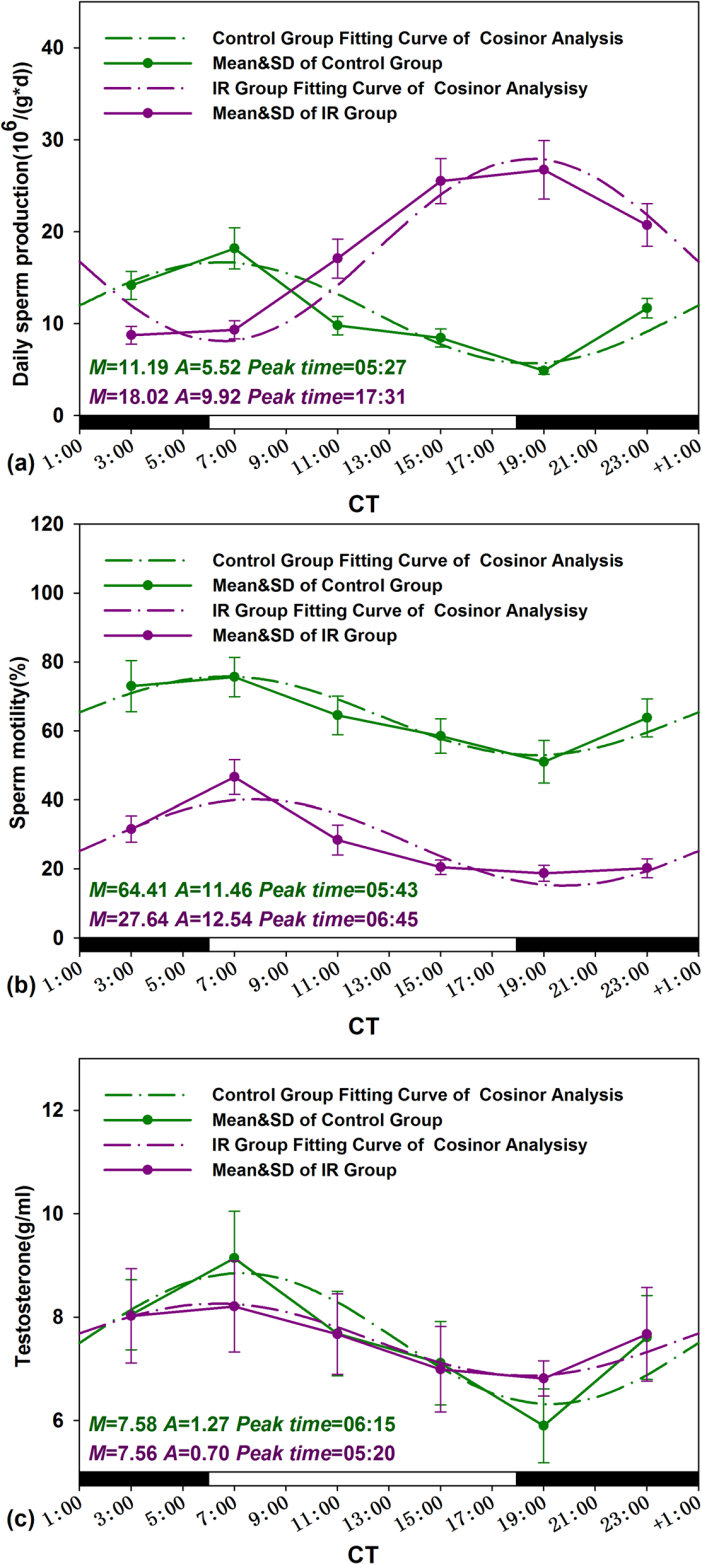
Circadian rhythm of reproductive markers of Control and IR mice. The best-fitting curves (means ± standard error) determined for DSP (**a**), sperm motility (**b**), and testosterone (**c**); *Y*-axis represents DSP, sperm motility and testosterone levels; *x*-axis represents the time during the 24-h light-dark cycle; *M* and *A* represents the median and the amplitude of the rhythm, respectively. Mice were exposed to X-ray (3 Gy) at CT 3:00, CT 7:00, CT 11:00, CT 15:00, CT 19:00, and CT 23:00 in a 24-h CT period. Control mice were in the same experimental circumstances without being exposed to X-ray. The white and dark boxes on the *x*-axis represent light and dark

**Fig. 4:**
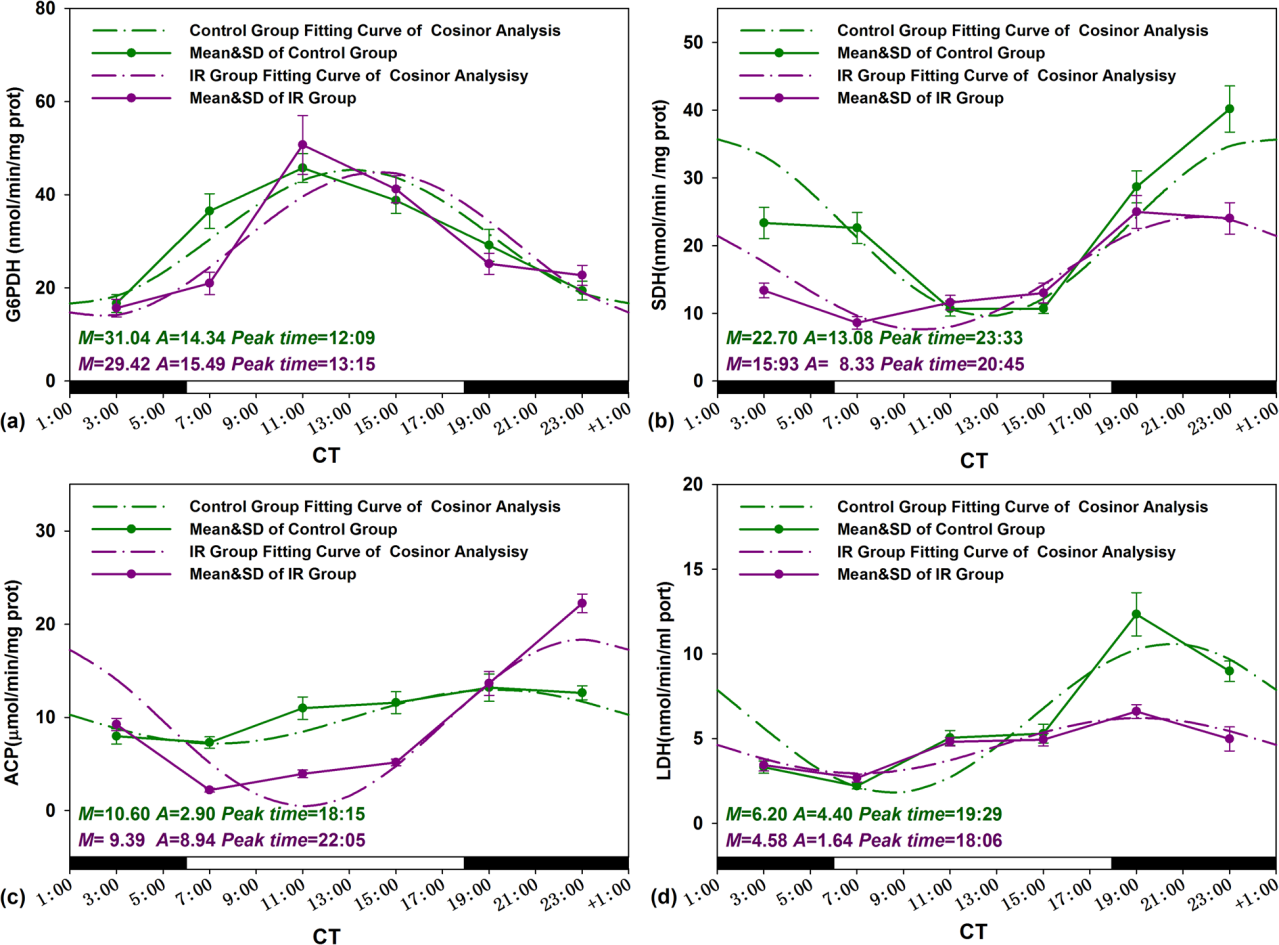
Circadian rhythm of testis mark enzymes activities of Control and IR mice. The best-fitting curves (means ± standard error) determined for G6PDH (**a**), SDH (**b**), ACP (**c)**, and LDH (**d**); *Y*-axis represents testis mark enzymes activities; *x*-axis represents the time during the 24-h light-dark cycle; *M* and *A* represents the median and the amplitude of the rhythm, respectively. Mice were exposed to X-ray (3 Gy) at CT 3:00, CT 7:00, CT 11:00, CT 15:00, CT 19:00, and CT 23:00 in a 24-h CT period. Control mice were in the same experimental circumstances without being exposed to X-ray. The white and dark boxes on the *x*-axis represent light and dark

Zero-amplitude test results of cosinor analysis of reproductive function data obtained from the control group were presented in Fig. 2a, b, and the straight lines and the cosinor fitting curves were presented in Figs. 3 and 4. By using zero-amplitude tests, the circles of confidence interval being not over-lapping with the pole (zero-point) suggested distinct circadian rhythms for the levels of three reproductive markers DSP, sperm motility, and testosterone (Fig. 2a), and the activities of four testicular marker enzymes ACP, G6PDH, SDH and LDH (Fig. 2b). From the fitting curves of cosinor analysis and the rhythm parameters in Figs. 3 and 4, the peak levels of DSP, sperm motility, testosterone, G6PGH, SDH, ACP, and LDH appeared at CT 5:27, CT 5:43, CT 6:15, CT 12:09, CT 23:33, CT 18:15, and CT 19:29, correspondingly.

**Fig. 5:**
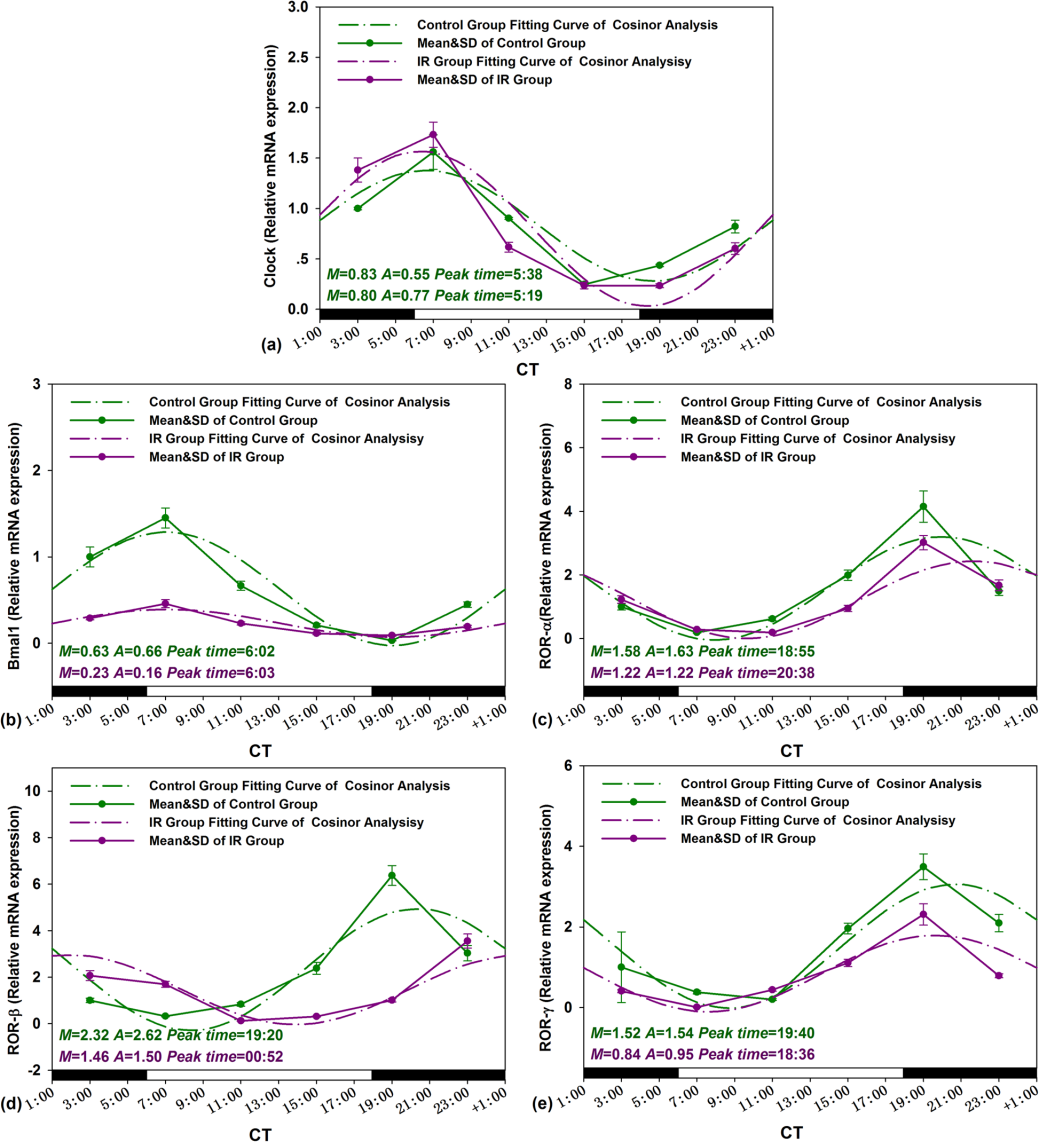
Circadian rhythm of testis clock genes expression levels of Control and IR mice. The best-fitting curves (means ± standard error) determined for *Clock* (**a**), *Bmal1* (**b**), *Ror-α* (**c)**, *Ror-β* (**d**), and *Ror-γ* (**e**); *Y*-axis represents mRNA expression level of clock genes; *x*-axis represents the time during the 24-h light-dark cycle; *M* and *A* represents the median and the amplitude of the rhythm, respectively. Mice were exposed to X-ray (3 Gy) at CT 3:00, CT 7:00, CT 11:00, CT 15:00, CT 19:00, and CT 23:00 in a 24-h CT period. Control mice were in the same experimental circumstances without being exposed to X-ray. The white and dark boxes on the x-axis represent light and dark

The same analysis was used for clock gene temporal mRNA expression (*Clock*, *Bmal1*, *Ror-α*, *Ror-β* and *Ror-γ*), the zero-amplitude test results revealed that the confidence interval circles of were not overlapped with the pole (Zero-point) to reject the zero-amplitude hypothesis, which indicated distinct rhythmic expression for these clock genes in mouse testis (Fig. 2c, d). Cosinor analysis showed the peak levels of *Clock*, *Bmal1*, *Ror-α*, *Ror-β*, and *Ror-γ* were at CT 5:38, CT 6:02, CT 18:55, CT 19:20, and CT 19:40, respectively (Fig. 5).

### Alteration in amplitude and peak time of reproductive markers and clock gene expressions induced by IR

The straight lines and the cosinor fitting curves of reproductive markers and clock genes expression obtained from control and IR two groups were presented in Figs. 3, 4, and 5. Amplitude and peak time are the important parameters of daily rhythm oscillations period. As showed in Fig. 3a, the oscillation phase of DSP in IR treated mouse testes were antiphase to the control, the peak time of DSP was shifted from CT 5:27 to CT 17:31 after radiation exposure. Meanwhile, the amplitudes of DSP oscillations period became severe in IR exposed testes from 5.52 to 9.95 (*p*<0.05). In contrast to the interconversion of DSP circadian phases in irradiated mice testis, the IR exposure does not affect significantly the phase distribution of other reproductive markers. Moreover, the amplitudes of testosterone levels and two testicular marker enzymes activities (SDH and LDH) were sharply shrunk after irradiation except ACP amplified (*p*<0.05) (Figs. 3c and 4b–d).

For circadian rhythms of clock genes expression, the peak time shifted more than one hour only happened in *Ror-α*, *Ror-β* expression after IR exposure (Fig. 5c, d), the amplitudes shrunk or amplified significantly in all five clock genes expression (*p*<0.05) (Fig. 5).

### Alteration in daily average level of reproductive markers and clock gene expressions induced by IR

The variation in the daily average levels of reproduction markers and clock genes in IR exposed mice was calculated using the formula: = ((Daily mean _IR_ – Daily mean _Control_)/Daily mean _Control_) × 100%. The results were presented in Table 5. IR exposure induced the changes in the daily mean values of the mice reproduction markers (including DSP, sperm motility, testosterone and G6PDH, SDH, ACP, and LDH) by 61.04%, − 57.09%, − 0.26 %, − 5.22%, − 29.82%, − 11.42%, and − 26.13%, respectively. For these reproductive parameters after IR treatment, the most remarkable daily mean variation was found in DSP (from 11.19 to 18.02 10^6^/g×d) and sperm motility (from 64.41 to 27.64 %) (Fig. 3a, b). However, IR treatment made effective sperm number (DSP multiply by motility) reduced observably from 7.21 to 4.98 10^6^/g×d.

**Table 5:** Percentage changes in the daily mean of male reproduction markers and clock genes expression between control and IR mice

Indicators	Daily mean changes (%)
**Daily sperm production**	**61.04**
**Motility**	**− 57.09**
**Testosterone**	**− 0.26**
**G6PDH**	**− 5.22**
**SDH**	**− 29.82**
**ACP**	**− 11.42**
**LDH**	**− 26.13**
***Clock***	**− 3.61**
***Bmal1***	**− 63.49**
***ROR-α***	**− 22.78**
***ROR-β***	**− 37.07**
***ROR-γ***	**− 44.74**

Note: (%) = ((Daily mean _IR_ – Daily mean _Control_)/Daily mean _Control_) × 100%

The alteration of daily average levels in gene expression of clock genes such as *Clock*, *Bmal1*, *Ror-α*, *Ror-β*, and *Ror-γ* in exposed mice after IR is presented in Table 5. The percentage changes of daily mean values of the clock genes expression happened in IR exposure mice by − 3.61% (*Clock*), − 63.49% (*Bmal1*), − 22.78% (*Ror-α*), 37.07 (*Ror-β*), and − 44.74% (*Ror-γ*), respectively. For the several clock genes, the most remarkable daily mean variation induced by IR treatment was found in *Bmal1* expression from 0.63 to 0.23 (Fig. 5b).

### Circadian sensitivity of reproductive markers and clock gene expressions to IR

As showed in Table 6, IR induced the different extent effects at six different circadian times: CT 3:00, CT 7:00, CT 11:00, CT 15:00, CT 19:00, or CT 23:00, respectively. Of the seven reproductive markers, the five parameters were increased or decreased at different timepoints, except for sperm motility and LDH activities going down at all circadian timepoints. The most significant change of these reproductive markers happened by − 68.37% for sperm motility, 447.30% for DSP, 15.52% for testosterone, − 42.45% for G6PDH, − 61.94% for SDH, 76.05% for ACP, and − 63.68% for LDH at CT 23:00, CT 19:00, CT 19:00, CT 7:00, CT 7:00, CT 23:00, and CT 19:00, respectively. For diurnal rhythm effect of clock genes, mRNA expression of *Clock* and *Bmal1* most prominently changed by IR at CT 19:00 by − 46.33% and 206.90%, respectively. The biggest variation of the other three clock gene expression induced by IR appeared at CT 11:00 for *Ror-a*, CT 7:00 for *Ror-β*, and CT 11:00 for *Ror-γ* by − 69.09%, 430.00%, and 120.10%, respectively (Table 6). The results showed that both male reproductive parameters and clock gene expression had their sensitive time points to IR.

**Table 6:** Circadian effects of IR on reproductive markers and clock genes expression

CT time	DSP	Sperm motility	Testosterone	G6PDH	SDH	ACP	LDH	***Clock***	***Bmal***	***ROR-a***	***ROR-β***	***ROR-γ***
**3:00**	**− 38.23**	**− 56.82**	**− 0.30**	**− 5.83**	**− 42.72**	**15.95**	**− 6.31**	**38.10**	**− 71.20**	**22.70**	**106.60**	**− 59.40**
**7:00**	**− 48.67**	**− 38.39**	**− 10.23**	**− 42.45**	**− 61.94**	**− 69.99**	**− 1.59**	**11.09**	**− 68.50**	**46.11**	**430.00**	**− 98.15**
**11:00**	**74.36**	**− 56.00**	**− 0.14**	**10.87**	**8.47**	**− 64.20**	**− 39.54**	**− 31.67**	**− 65.56**	**− 69.09**	**− 85.82**	**120.10**
**15:00**	**201.89**	**− 65.01**	**− 1.69**	**6.21**	**21.50**	**− 55.55**	**− 32.91**	**− 4.45**	**− 45.89**	**− 52.43**	**− 87.02**	**− 43.52**
**19:00**	**447.30**	**− 63.35**	**15.52**	**− 13.63**	**− 12.93**	**3.42**	**− 63.68**	**− 46.33**	**206.90**	**− 27.21**	**− 84.21**	**− 33.83**
**23:00**	**77.36**	**− 68.37**	**0.80**	**16.90**	**− 40.17**	**76.05**	**− 23.35**	**− 26.55**	**− 57.05**	**12.06**	**17.19**	**− 62.34**

Note: (%) = ((Mean IR CTx:00 – Mean control CTx:00)/Mean control CTx:00) × 100%

## Discussion

The mammalian circadian clock controls the timing of many biological processes. Reproductive physiology, particularly, is profoundly under the circadian regulation [21, 22]. Environmental cues such as light exposure can influence the timing of this internal clock system through the post translational modification of key components of the core molecular oscillator. During deep space exploration, radiation is one of the major challenges to astronauts. We have previously shown that radiofrequency radiation exposure can reset the circadian clock of the reproductive system in a time-of-day-dependent manner in male rats [12, 23]. However, the potential effects of space IR on the circadian rhythms of male reproductive functions have not been well characterized so far. In the present study, we found a dramatic reproductive disruption concurrent with the decreased daily mean expression levels of five clock genes in the mice testes exposed to 3 Gy IR. The reproductive disruptions were induced by IR, in terms of reversed DSP rhythm, and lower amplitudes of testosterone level, decreased SDH and LDH activities, and down-regulation of clock gene expressions. Furthermore, a significant timing effect of IR exposure on reproductive functional markers and clock gene expression was observed, when the changes between the IR exposed group and its sham control exposed group were compared at a specific corresponding time-point. These observations suggest that IR exposure can disrupt the reproductive function through resetting its circadian clock in mouse testis, indicating a significant role of the circadian clock in mediation of reproductive toxic effects of IR exposure.

Earlier studies have reported inconsistent results in the circadian expression of clock genes in normal mice testicles. While a nonrhythmic and developmental-stage specific expression of *Cry1*, *Clock*, and *Ror-α* in mice testicles was observed, more recent studies have revealed rhythmic expression of *Per1*, *Per2*, and *Bmal1* genes in the testis of hamsters and mice [21, 24]. In agreement with these studies, our results showed the diurnal periodicity of productive functions in mice, with their level peak times at CT 5:27, CT 5:43, and CT 6:15 for three male reproductive markers (DSP, sperm motility, and testosterone, respectively), and the four enzymes (G6PGH, SDH, ACP, and LDH) activities peak times at CT 12:09, CT 23:33, CT 18:15, and CT 19:29, respectively. To further understand whether the daily variation of male reproductive function is correlated with inherent rhythmic expression of clock genes in mouse testis, we examined the kinetics of clock gene expression in mRNA level and found that the expressions of all the five clock genes were oscillating throughout the day, with peaking times of *Clock* and *Bmal1* gene at CT 5:38 and CT 6:02, and peaking times of *Ror-α*, *Ror-β*, and *Ror-γ* gene at CT 18:55, CT 19:20, and CT 19:40, respectively.

Ionizing radiation has been shown to induce tissue injury during space exposure to SPEs [25]. In this study, the simulated dose of 3 Gy IR obviously altered the daily mean level of sperm motility, a male reproductive marker that may be resulted from radiation-induced apoptosis and inflammation of the seminiferous tubules [7, 10, 26–32]. Spermatogenesis is a high energy demanding process, and the activities of energy metabolism-related enzymes, including SDH and LDH, play key roles for the growth and development of testicular cells. For example, SDH, which is mainly found in chondriosome of Sertoli cells and spermatogenic cells, functions in pachytene spermatocyte maturation of germinal epithelium [33]. SDH is also responsible for providing energy to sperm cells by converting sorbitol to fructose. Similarly, LDH in Sertoli and spermatogenic cells also takes part in testis energy production and biotransformation. ACP, a lysosomal original enzyme in Sertoli cells of seminiferous tubules, has been reported to be involved in the development of spermatocytes and steroidogenesis, while G6PDH activities parallel the synthesis function of steroid hormone in Leydig cells [19, 34]. In this study, the daily mean activities of G6PDH, SDH, LDH, and ACP were decreased after IR exposure, indicating the disruption in male reproductive functions induced by IR exposure.

There are many reports that the clock genes are involved in regulating male reproductive functions. Our previous work has shown that one of the clock genes, *Ror-α*, plays a key role in testosterone biosynthesis through the StAR-P450 signal axis [12]. Epidemiological investigations have indicated associations of genetic variability in the *Clock* gene with semen quality in idiopathic infertile men [35] and increased risk for male infertility [36, 37]. Mutations in the *Clock* gene can result in reduced male fertility in mouse [38, 39], and mutations in the *Bmal1* gene altered the rate-limiting step of steroidogenesis that is a prerequisite for sperm production and maturation [40]. Thus, it is interesting to investigate whether IR-induced disruption of reproductive function is associated with altered clock gene expression. In this study, we demonstrated that the daily mean mRNA levels of all the five tested clock genes were significantly modified by IR exposure, which is consistent with the concurrent downregulation of reproductive functional marker enzymes in irradiated mice. These data raise the possibility that IR-induced suppression in daily mean expression of clock genes may be accounted for the subsequent reproductive function inhibition in mouse testes.

By using genetic circadian mutant mice models, recent studies have shown that the clock system is responsible to genotoxic stresses [41–43]. In support of these findings, IR exposure resulted in disrupted circadian oscillations in both DSP activity and clock gene expression level (*Ror-α* and *Ror-β*, indicated by peak time). These results reinforce the concept that for the clock to regulate reproductive functions, it must be responsive to environmental stress in time to optimally coordinate cellular metabolism with externally fluctuating metabolic demands [44, 45].

Our previous work has found that the circadian clock can significantly alter the DSP and sperm motility of rat, as well as the levels of antioxidants such as glutathione peroxidase (GSH-Px), superoxide dismutase (SOD), and melatonin, in response to radiofrequency radiation [23, 46]. To follow up the work, this study is to make further efforts to explore the circadian effect of IR on reproductive functions, by treatment of mice to X-ray irradiation at six different time points during the day (CT 3:00, CT 7:00, CT 11:00, CT 15:00, CT 19:00, and CT 23:00). As expected, our results did show some differential effects of IR at various circadian times. For example, changes in sperm motility and DSP were most significant in mice irradiated at CT 23:00 and CT 19:00, respectively, and the gene expressions of *Ror-β* and *Bmal1* were most upregulated in the same groups of mice irradiated at CT 7:00 and CT 19:00, respectively. These results indicated that not only IR itself, but the daily time at which radiation is exposed, may have a significant impact on the reproductive functions in a time-dependent manner.

## Conclusion

Taken together, the present study provides with evidence of circadian adverse effects of IR on reproductive functions in male mouse. These effects are present in terms of both average daily levels of the reproductive markers, and circadian oscillation profiles of the clock genes expressions, which may be of value in the protection of astronauts in deep space exploration.

## Data Availability

All data generated or analyzed during this study are included in this published article.
